# Molecular and Growth-Based Drug Susceptibility Testing of* Mycobacterium tuberculosis* Complex for Ethambutol Resistance in the United States

**DOI:** 10.1155/2016/3404860

**Published:** 2016-06-08

**Authors:** Mitchell A. Yakrus, Jeffrey Driscoll, Allison McAlister, David Sikes, Denise Hartline, Beverly Metchock, Angela M. Starks

**Affiliations:** Centers for Disease Control and Prevention, Atlanta, GA 30329-4027, USA

## Abstract

Ethambutol (EMB) is used as a part of drug regimens for treatment of tuberculosis (TB). Susceptibility of* Mycobacterium tuberculosis* complex (MTBC) isolates to EMB can be discerned by DNA sequencing to detect mutations in the* embB* gene associated with resistance. US Public Health Laboratories (PHL) primarily use growth-based drug susceptibility test (DST) methods to determine EMB resistance. The Centers for Disease Control and Prevention (CDC) provides a service for molecular detection of drug resistance (MDDR) by DNA sequencing and concurrent growth-based DST using agar proportion. PHL and CDC test results were compared for 211 MTBC samples submitted to CDC from September 2009 through February 2011. Concordance between growth-based DST results from PHL and CDC was 88.2%. A growth-based comparison of 39 samples, where an* embB* mutation associated with EMB resistance was detected, revealed a higher percentage of EMB resistance by CDC (84.6%) than by PHL (59.0%) which was significant (*P* value = 0.002). Discordance between all growth-based test results from PHL and CDC was also significant (*P* value = 0.003). Most discordance was linked to false susceptibility using the BACTEC*™* MGIT*™* 960 (MGIT) growth-based system. Our analysis supports coalescing growth-based and molecular results for an informed interpretation of potential EMB resistance.

## 1. Introduction

In 2014, 9,412 new tuberculosis (TB) cases were reported in the United States [1]. Of these cases, 96 (1.3%) were classified as multidrug resistant (MDR), defined as resistance to at least rifampin (RMP) and isoniazid (INH). Reliable drug susceptibility testing (DST) for isolates of* Mycobacterium tuberculosis* complex (MTBC) is essential for selection of effective treatment regimens, interruption of transmission, and prevention of further development of resistant forms of TB.

Ethambutol (EMB) in combination with INH, RMP, and pyrazinamide (PZA) is used as part of a first-line antituberculosis drug regimen for patients with drug-susceptible TB. EMB is often included, in combination with second-line drugs, as part of the treatment regimen for MDR-TB when the isolate is susceptible [2–4]. EMB is a bacteriostatic antimicrobial that interferes with cellular metabolism by inhibition of arabinosyltransferase required for biosynthesis of arabinogalactan in the cell wall [4, 5]. Mutations at the* embCAB* operon, which encode mycobacterial arabinosyltransferase, are significantly associated with growth-based resistance to EMB [6]. These mutations are most frequently reported at either* embB* codon 306 or* embB* codon 406 [7–9].

Nonsynonymous mutations have been detected at other codons outside these locations between codons 296 and 497 in EMB-resistant isolates [10]. However, mutations reported at these codons, such as Glu378Ala, may be lineage markers not associated with resistance [11–14]. Therefore, DNA sequencing alone cannot be relied upon to detect EMB resistance due to the presence of mutations not conferring growth-based resistance and because other mechanisms of EMB resistance may exist [13, 14]. Discordant results among test methods of growth-based DST for EMB resistance have been well documented and linked to difficulties establishing equivalent critical concentrations (CC) [7, 15–19]. In addition, allelic exchange experiments have demonstrated that some* embB* 306 mutations, such as Met306Ile, may result in only a moderately raised minimal inhibitory concentration (MIC) above the CC and MTBC isolates with these mutations may be falsely reported as susceptible [8, 20]. Both EMB-susceptible and EMB-resistant isolates with Met306Ile mutations were reported in the same study where agar proportion was used for growth-based DST [13].

The Centers for Disease Control and Prevention (CDC) provides molecular detection of drug resistance (MDDR) through DNA sequencing of loci associated with TB-drug resistance, including EMB resistance, and concurrent growth-based DST. Molecular testing can be performed with either MTBC isolates or sediments of clinical specimens that are positive for MTBC by nucleic acid amplification tests (NAAT) [13]. This service is available upon request by public health laboratories (PHL) for samples meeting defined submission criteria [21]. PHL submitting MTBC samples for testing receive an interim report with molecular results and a final report upon completion of growth-based DST. The final report contains interpretive comments based on both molecular and growth-based results. CDC's MDDR service has been described previously [22, 23].

Previously, we examined the concordance between molecular and growth-based DST for detection of RIF and INH resistance of MTBC samples submitted to CDC's MDDR service [23]. In this study, we compared EMB susceptibility results from the MDDR service, molecular and growth-based, with growth-based results provided by PHL. In addition, we analyzed test results and methods for probable causes of discordance.

## 2. Materials and Methods

### 2.1. MTBC Samples and Collection of Growth-Based DST Results from PHL

EMB test results analyzed for this study were MTBC isolates and NAAT-positive sediments from TB patients submitted by PHL to CDC's MDDR service from September 2009 to February 2011. Growth-based DST results and test methods used for these samples at PHL were available from a previously described study that used a secure survey instrument to collect data online from PHL [23, 24]. CDC determined that the prior study was not human subjects' research; thus, it did not require Institutional Review Board review. Growth-based DST results for EMB were successfully collected for 211 MTBC samples submitted by PHL during the study timeframe. Collection of all data was approved under an Office of Management and Budget (OMB) generic clearance package (Information Collections to Advance State, Tribal, Local and Territorial Governmental Agency System Performance, Capacity, and Program Delivery; OMB number 0920-0879) as required under the Paperwork Reduction Act.

### 2.2. Growth-Based DST and DNA Sequencing

Growth-based DST for EMB was performed at CDC using the indirect agar proportion method using a critical concentration (CC) of 5 *μ*g/mL in supplemented Middlebrook 7H10 agar [25]. PHL performed growth-based DST on 211 MTBC samples submitted to CDC's MDDR using either BACTEC MGIT 960 (MGIT) system (Becton Dickinson and Company) (136 samples), BACTEC 460 (Becton Dickinson and Company) (45 samples), BACTEC 460 and agar proportion (18 samples), agar proportion (2 samples), VersaTrek*™* (Trek Diagnostic Systems) (1 sample), or isolates that were referred to another laboratory (9 samples) where the DST method was unknown. DNA sequencing for detection of mutations at the* embB* locus associated with EMB drug resistance was performed as previously described [13].

### 2.3. Data Analysis

Growth-based DST data from PHL were analyzed using PASW Statistics (version 18; IBM SPSS software). Concordance between testing at CDC (both DNA sequencing and growth-based DST) and growth-based testing performed by PHL was determined by cross-tabulation of results and calculation of percent agreement. Sample proportions were compared using McNemar's test without continuity correction with a significance level of *P* value = 0.05.

## 3. Results and Discussion

### 3.1. Comparison of Growth-Based DST Performed by PHL with DNA Sequencing and Growth-Based DST Performed by CDC

The cross-tabulation of results for determination of EMB resistance from growth-based DST from PHL and from DNA sequencing and growth-based DST performed by CDC is shown in Table 1. Of the 211 MTBC samples submitted by PHL with a corresponding growth-based DST result for EMB, a growth-based DST result was not available for comparison from 30 samples tested by CDC. Absence of growth-based DST results was due to either contamination (14 samples) or failure to grow (16 samples). DNA sequencing was not performed at CDC for 12 samples submitted in 2009 before molecular testing for EMB resistance was added. CDC detected 14 MTBC samples that contained either Glu378Ala or Leu355Leu neutral polymorphisms confirmed to be EMB-susceptible by CDC agar proportion. PHL growth-based EMB results and DNA sequencing and growth-based results from CDC were available for comparison for 170 of the 211 MTBC samples listed in Table 1. There was agreement between growth-based DST results from both PHL and CDC for 150 samples resulting in an overall agreement of 88.2%.

Cross-tabulation of whether or not an* embB* mutation associated with EMB resistance was detected using DNA sequencing of MTBC samples by CDC with the number resistant by growth-based DST at both PHL and CDC is shown in Table 2. DNA sequencing determined that 39 samples (22.9%) of the 170 MTBC samples with growth-based results available from both PHL and CDC contained an* embB* mutation associated with resistance. When an* embB* mutation associated with resistance was detected, a higher percentage (84.6%) of these samples were found to be resistant using growth-based DST at CDC by agar proportion compared with growth-based DST performed by PHL (59.0%), and this difference was significant (*P* value = 0.002). There was no significant difference in growth-based DST results between CDC and PHL for MTBC samples where no mutation was detected (*P* value = 0.317). However, for all 170 MTBC samples examined, there was a significant difference (*P* value = 0.003) between growth-based determination of EMB resistance performed by PHL and that performed by CDC.

### 3.2. Discordance between Growth-Based DST Performed by PHL and CDC

Discordant results between PHL and CDC including DST methods used are listed in Table 3. There were 20 discordant test results between growth-based DST performed by PHL and agar proportion performed by CDC of which 16 (80%) samples were found to be susceptible to EMB by PHL and resistant to EMB by CDC. The growth-based DST method most frequently used by PHL among these 16 samples was MGIT (11 samples). For 10 of these 16 samples, testing by CDC detected* embB* mutations associated with EMB resistance at either codon 306 (6 samples), codon 406 (3 samples), or codon 296 (1 sample). For the six other discordant results in this category, DNA sequencing by CDC did not detect an* embB* mutation. However, it has been reported that MTBC isolates may be EMB-resistant using agar proportion without molecular detection of an* embB* mutation [13, 26]. For three of the MTBC samples with discordant results, PHL reported EMB resistance using MGIT while molecular testing by CDC did not detect an* embB* mutation and these samples were susceptible using agar proportion. PHL also reported EMB resistance using MGIT for one sample where molecular testing at CDC detected a mutation not associated with* embB* resistance at codon 378 (Glu378Ala) and found it to be EMB-susceptible by agar proportion.

Combined molecular and growth-based test results from CDC suggest that most discordance with PHL growth-based DST was due to false susceptibility to EMB. False susceptibility to EMB may occur for various reasons. Some EMB-resistant strains grow better on solid media versus liquid media (such as media used with the MGIT system) [16, 18]. Therefore, even though the recommended CC for determining primary resistance to EMB for MGIT and agar proportion are both 5 *μ*g/mL, these test concentrations may not be equivalent when comparing results using these test methods [27]. Specific mutations may affect the MIC of the isolate such that the variability around the CC is due to the MIC being close to the CC, thus affecting false susceptibility in MGIT. Heteroresistance may be present with late growth of resistant mutants on solid media in the presence of EMB with failure to detect these mutants in the liquid-based MGIT system due to lack of growth [17].

## 4. Conclusions

Most laboratories rely on a single growth-based DST method such as the well-established MGIT system. Though the MGIT system has been found to be reliable for growth-based DST of MTBC isolates for most antituberculosis drugs, this study and previous reports have found discrepant results when this method is used solely for determination of EMB resistance [16, 18, 19, 28]. By providing both molecular detection and growth-based DST by agar proportion, CDC's MDDR detected a significantly higher number of MTBC samples that were EMB-resistant than PHL that employed only growth-based methods. Our results reinforce the importance of combining molecular testing with a reliable method of growth-based DST for accurate detection of EMB-resistant TB.

## Figures and Tables

**Table 1:** Comparison of PHL growth-based DST with CDC's molecular detection and growth-based DST results for EMB.

PHL growth-based DST result for EMB	CDC molecular result for *embB* (amino acid change)	CDC's agar proportion result (number of MTBC samples)				
		Resistant	Susceptible	No growth	Contaminated	Total number of samples
Resistant	Met306Ile	4	0	3	0	7
	Met306Ile, Asp328Gly	0	0	1	0	1
	Met306Ile, Gly406Ala	1	0	0	0	1
	Met306Val	9	0	3	1	13
	Phe330Leu	1	0	0	0	1
	Tyr334His	2	0	0	0	2
	Ser347Thr	1	0	0	0	1
	Asp354Ala	2	0	1	0	3
	Glu378Ala	0	1	0	0	1
	Gly406Ala	2	0	0	0	2
	Gly406Asp	1	0	0	0	1
	No mutation	4	3	0	1	8
	Not performed	2	0	0	0	2

Susceptible	Gly294Gly	0	1	0	0	1
	Asn296Tyr	1	0	0	0	1
	Met306Ile	2	1	1	0	4
	Met306Val	4	0	0	0	4
	Val309Ile	0	0	1	0	1
	Leu355Leu	0	1	0	0	1
	Leu355Leu, Glu378Ala	0	3	0	0	3
	Glu378Ala	0	9	4	2	15
	Gly406Ala	0	2	0	0	2
	Gly406Cys	2	1	0	0	3
	Gly406Ser	1	0	0	0	1
	No mutation	6	105	2	9	122
	Not performed	0	9	0	1	10

Total		45	136	16	14	211

PHL: public health laboratory; DST: drug susceptibility testing; CDC: Centers for Disease Control and Prevention; EMB: ethambutol; MTBC: *Mycobacterium tuberculosis* complex.

**Table 2:** Cross-tabulation of CDC's molecular detection with both PHL and CDC growth-based DST results for determination of EMB resistance.

Detection of *embB* mutation by CDC's MDDR	Number of samples	Number of EMB-resistant MTBC samples (%)		*P* value
		PHL growth-based DST	CDC agar proportion	
Yes	39	23 (59.0)	33 (84.6)	0.002
No^a^	131	7 (5.34)	10 (7.63)	0.317

Total	170	30 (17.6)	43 (25.3)	0.003

^a^Including samples with Glu378Ala and Leu355Leu polymorphisms not associated with EMB resistance.

CDC: Centers for Disease Control and Prevention; PHL: public health laboratory; DST: drug susceptibility testing; EMB: ethambutol; MTBC: *Mycobacterium tuberculosis* complex; MDDR: molecular detection of drug resistance.

**Table 3:** Summary of 20 discordant results for detection of EMB resistance between PHL and CDC.

Number of samples	PHL growth-based DST result	PHL growth-based DST method	*embB* mutation detected by CDC's MDDR	CDC's agar proportion result
1	Susceptible	BACTEC 460 and agar proportion	Asn296Tyr	Resistant
1	Susceptible	MGIT 960	Met306Ile	Resistant
1	Susceptible	BACTEC 460 and agar proportion	Met306Ile	Resistant
3	Susceptible	MGIT 960	Met306Val	Resistant
1	Susceptible	BACTEC 460	Met306Val	Resistant
1	Resistant	MGIT 960	Glu378Ala	Susceptible
2	Susceptible	MGIT 960	Gly406Cys	Resistant
1	Susceptible	Not performed in-house	Gly406Ser	Resistant
3	Resistant	MGIT 960	None	Susceptible
5	Susceptible	MGIT 960	None	Resistant
1	Susceptible	Not performed in-house	None	Resistant

PHL: public health laboratory; DST: drug susceptibility testing; CDC: Centers for Disease Control and Prevention; MDDR: molecular detection of drug resistance.
